# Pedunculated lipoma causing colo-colonic intussusception: a rare case report

**DOI:** 10.1186/1471-2482-13-51

**Published:** 2013-10-30

**Authors:** Ouadii Mouaqit, Hafid Hasnai, Leila Chbani, Abdelmalek Oussaden, Khalid Maazaz, Afaf Amarti, Khalid Ait Taleb

**Affiliations:** 1 Surgery Departement, University Hospital Hassan II, BP 1893; Km 2.200, Sidi Harazem Road, Fez 30000, Morocco; 2 Departement of Pathology, University Hospital Hassan II, BP 1893; Km 2.200, Sidi Harazem Road, Fez 30000, Morocco; 3 46, Avenue Ibn el Khatib, Immeuble 46, Lotissement Ghazali, Quartier elAzhar, Fes, Morocco

**Keywords:** Intussusception, Lipoma, Abdominal computed tomography, Colo-colic, Invagination

## Abstract

**Background:**

Intussusception is a relatively common cause of intestinal obstruction in children but a rare clinical entity in adults, representing fewer than 1% of intestinal obstructions in this patient population. Colonic lipomas are uncommon nonepithelial neoplasms that are typically sessile, asymptomatic and incidentally found during endoscopy, surgery, or autopsy.

**Case presentation:**

A 55-year old man visited our emergency department with severe abdominal pain, multiple episodes of vomiting, abdominal distension. Abdominal ultrasound sonography and computed tomography showed a sausage-shaped mass presenting as a target sign, suggestive of intussusception. Surgery revealed a hard elongated mass in the right colon wihch telescoped in the transverse colon and caused colo-colonic intussusception. Rhigt hémicolectomy was performed and pathology documented a mature submucosal lipoma of the colon. We describe the difficulties in diagnosis and management of this rare cause of bowel obstruction and review the literature on adult intussusceptions.

**Conclusion:**

A large submucosal lipoma is a very rare cause of colon intussusception that presents as intestinal obstruction in patients without malignancy. CT and magnetic resonance imaging remain the methods of choice for studying abdominal lipomas, particularly those rising into the layers of the colonic wall. Surgical resection remains the treatment of choice and produces an excellent prognosis.

## Background

Intussusception was reported for the first time in 1674 by Barbette of Amsterdam. Intussusception is relatively frequent in children but is rare in adults [1]. Adult Intussusception represents 1% of all bowel obstructions and 5% of all bowel intussusceptions [1,2]. Lipoma and may develop as a benign tumor in all organs and rarely in large or small intestine. Gastrointestinal lipomas are rare benign tumors and intussusception due to a gastrointestinal lipoma constitutes an infrequent clinical entity [2]. Colonic lipoma typically presents as a sessile polypoid mass. Infrequently, lipomas of the colon are pedunculated, with ulcerated or necrotic overlying mucosa. We present an extremely rare case of a symptomatic pedunculated lipoma of the colon transverse with ulcerated mucosa causing intermittent colo-colonic intussusception that was surgically resected.

## Case presentation

**Figure 1:**
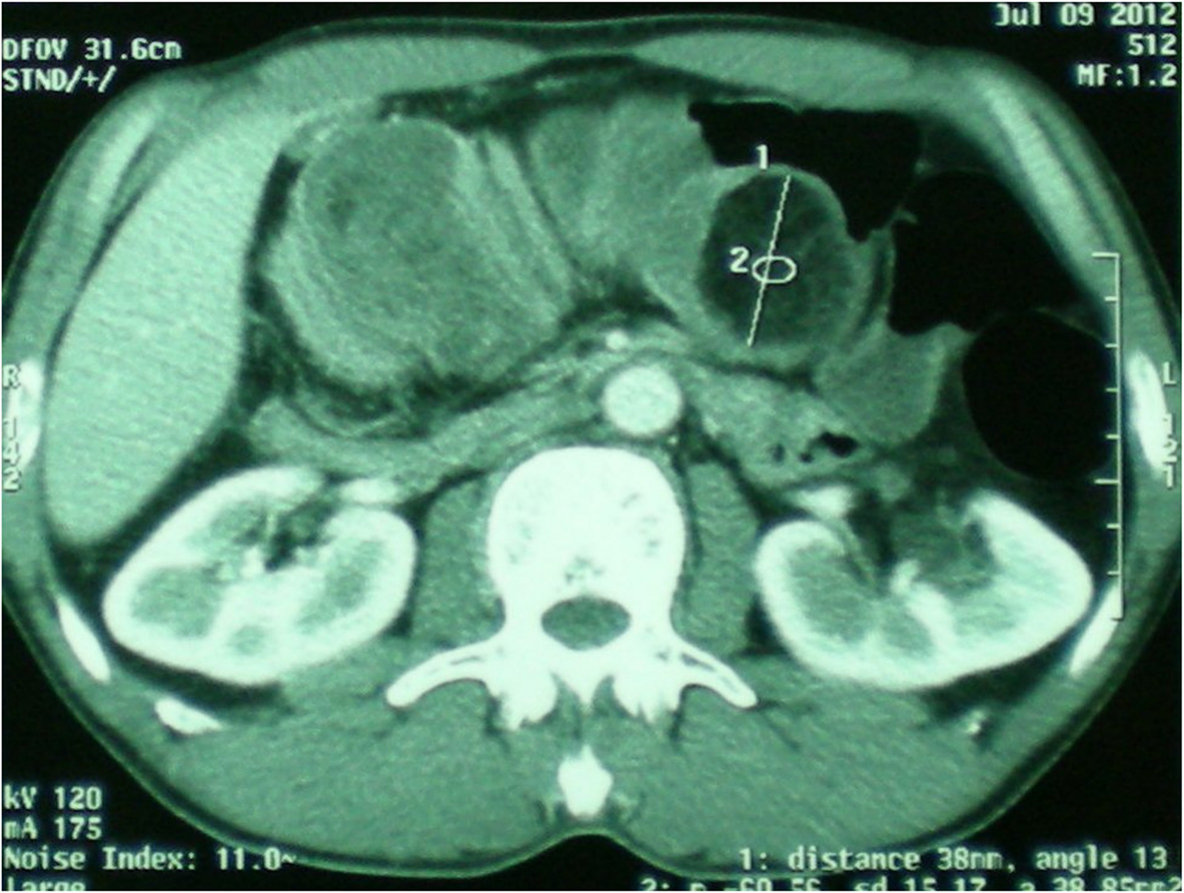
**CT scan showed findings indicative of an colon intussusception as a target-like mass.** A round mass of fat density representing lipoma was detected within the lumen of the intussuscipiens.

**Figure 2:**
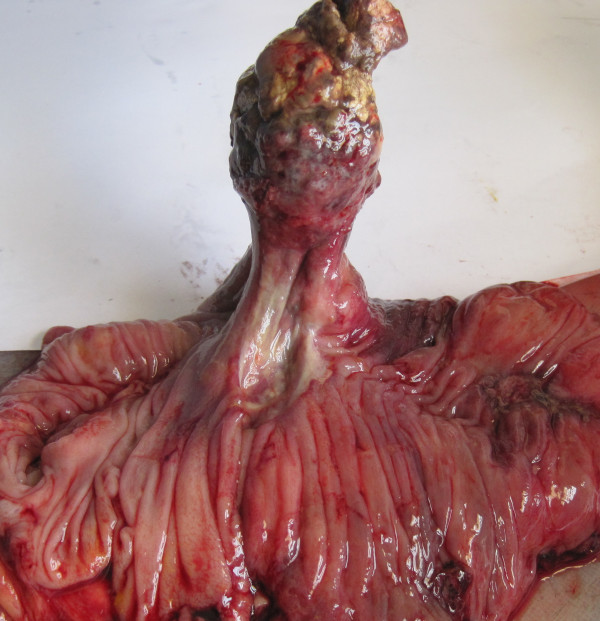
A pendulant polipoid submucosal tumor of the transverse colon served as a lead point for the intussusception.

**Figure 3:**
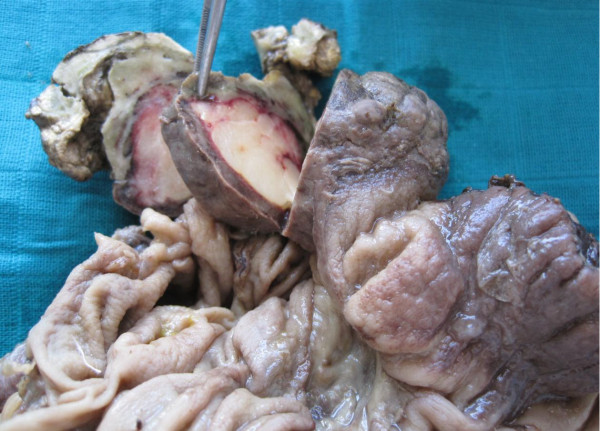
Resected specimen demonstrating fatty consistency.

**Figure 4:**
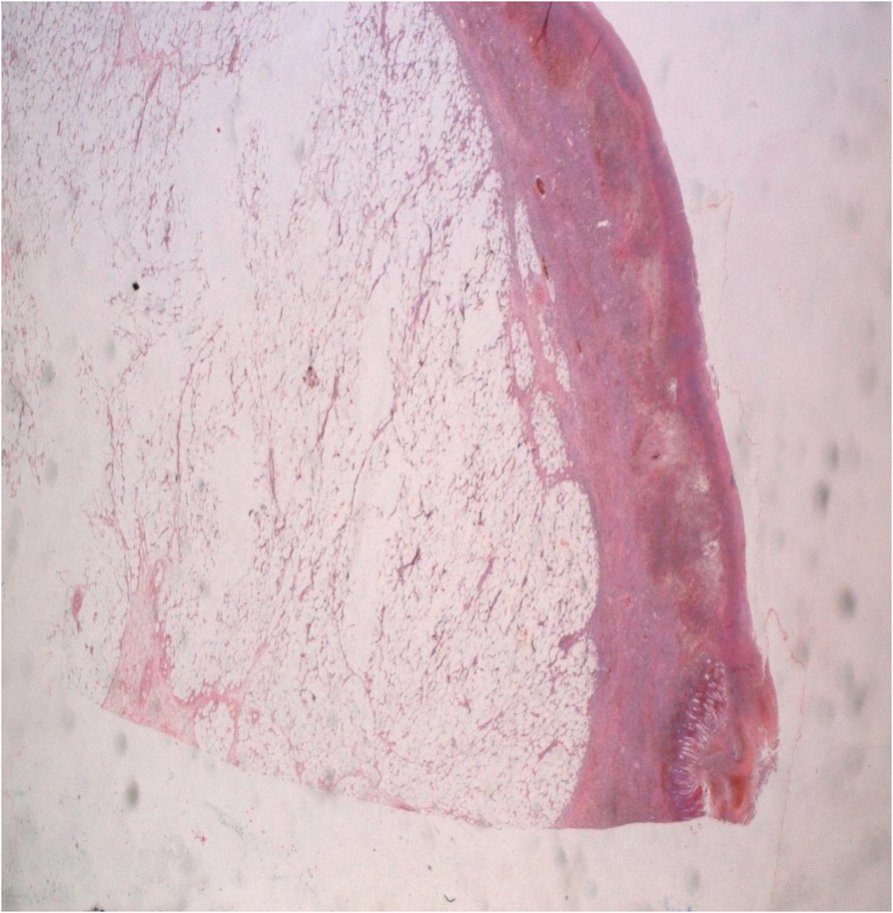
A histopathologic examination of the tumor revealed fat cells proliferating in the submucosal layer.

A 55-year old man presented with a three day history of colicky abdominal pain and bilious vomiting. The patient had no past history of peptic ulcer disease, alteration in bowel habits, melena or weight loss. On examination, he was apyrexial and hemodynamically stable. His abdomen was distended with localized tenderness in the right iliac fossa and no palpable abdominal masses; bowel sounds were hyperaudible. Initial A rectal examination revealed no masses or blood. The laboratory findings on admission were as follows: normal red blood cell count (4420000/mm), normal white blood cell count (10200/mm3), and normal platelet count (151000/mm3). The total protein, and serum creatinine levels were within the normal range, as were the serum carcinoembryonic antigen and cancer antigen19-9 levels. Abdominal CT showed a target sign- or sausage-shaped lesion typical of an intussusception that varied in appearance relative to the slice axis. More head-side scans showed a low-density homogenous mass measuring 3 cm that was considered to be the leading point for the invagination (Figure 1). These findings led to a diagnosis of intussusceptions induced by a lipoma. Colonoscopy was performed to assess the lesion further and attempt to reduce the intussusception however when this was not possible. The patient was transferred to the operating room for exploratory laparoscopy, which revealed the presence of a colo-colonic intussusception in the right colon. Because of compromised perfusion and swelling of his colonic wall and because of an unsuccessful attempt at manual desinvagination, a right hemicolectomy was performed. The continuity single-layer end-to-end ileotransverse anastomosis. The postoperative period was uneventful and the patient was discharged on the sixth postoperative day. Macroscopic assessment of the resected specimen showed the presence of a round pedunculated colonic polypoid tumor of 3 × 3 × 4,5 cm in size with the features of lipoma, causing intussusception of the ascending colon into transverse colon (Figures 2 and 3). The histological examination revealed mature fat cells, connective tissue, and scattered blood vessels within the removed submucosal mass (Figure 4). There was no evidence of dysplasia or malignancy.

## Conclusion

Intussusception remains a rare condition in adults, representing 1% of bowel obstructions or 0.003% to 0.02% of all hospital admissions. 90% of adult intussusceptions have an organic cause, 60% developing due to neoplasms (60% malign and 24–40% benign) [1]. Adult colonic intussusception is caused by a primary carcinoma in 65–70% of all cases. Colonic lipoma as leading cause is uncommon [3]. They are more common in women with a peak incidence between 50 and 60 years old [4,5]. Most are found in the cecum, located submucosally. Colonic lipoma typically presents as a sessile polypoid mass, arising from the submucosa with an intact mucosa. Infrequently, lipomas of the colon are pedunculated, with ulcerated or necrotic overlying mucosa. Several possible mechanisms have been proposed to explain this situation: (a) a tumor may act as a foreign body causing violent peristalsis, so that the contracted central part of the bowel easily moves into the dilated distal part; (b) intussusception may be due to altered muscle function caused by a tumor or bowel paralysis; and (c) a tumor may be grasped and pulled forward by traction [6,7]. The clinical presentation of intussusceptions can be very diverse in the adult [3,4,6]. Abdominal pain is the most common symptom followed by obstruction and palpable mass. Common symptoms are abdominal pain, vomiting, and bloody stools presenting for many days or even weeks. Physical examination may show a palpable “sausage-like” mass, and blood tests may show significant leucocytosis. Since only about 9% to 10% of adult intussusceptions present with the typical triad of abdominal pain, palpable abdominal mass and bloody stool. A study in the Mayo clinic showed that 94% of lipomas are asymptomatic however isolated lipomas may present with non specific abdominal pain, bleeding and constipation [5]. The preoperative diagnosis is usually very difficult [1,4,8]. Imaging modalities can contribute to the preoperative diagnosis of colonic lipomas. Barium enema usually reveals a filling defect; however this finding is no specific of colonic lipoma or any other type of colonic neoplasm. On CT a lipoma has a uniform appearance with fat-equivalent density and a smooth border(−40 till −120 Hounsfield units) [9]. However, even if the radiological image may suggest strong evidence for the existence of a lipoma, radiologists will often also mention other more malignant options in their differential diagnosis. Recent reports [10] in the literature have suggested that abdominal CT scanning is the preferred radiologic modality for diagnosing intussusception from colonic lipomas. The sensitivity of CT scan to correctly diagnose intussusceptions has been reported from 71.4%-87.5% while its specificity in adults has been reported to be 100% as verified by the subsequent surgery. For patients with features typical of colonic lipoma, CT reliably confirms the diagnosis. However, intussuscepted lipomas may not demonstrate normal fat attenuation and may have a heterogeneous appearance reflecting the degree of infarction and fat necrosis present at the time of radiologic evaluation [11]. Some investigators have recommended enteroclysis for intussusception diagnosis, but only one case has been reported [12]. Magnetic resonance imaging is particularly able to detect fatty lesions because of signal intensity characteristics typical for adipose tissue mainly on T1-weighted and fat-suppressed images. However, this imaging modality is seldom used for detecting and studying intestinal neoplastic lesions [10]. Colonoscopy can usually distinguish colonic lipomas from cancer and other neoplasias, especially when the overlying mucosa is intact. However it should be kept inmind that in few cases, accurate preoperative diagnosis can be difficult [13]. In view of the uncertain etiology and diagnosis and high incidence of malignancy (approaching 50%), the treatment of intussusception in adults is invariably surgical resection [2,4-6]. The majority of authors recommend surgery as the standard method of treatment for every colonic lipoma greater than 2 cm in size [4,6]. Surgical treatment includes resection, colotomy with local excision, limited colon resection, segmental resection, hemicolectomy, or subtotal colectomy. The choice of any of the above mentioned surgical interventions mainly depends on the lipoma size, location, and the presence or absence of definite preoperative diagnosis or disease complications. The time and the type of the surgical intervention differ and depend on the site, cause, and degree of obstruction. Most surgeons agree that resection is necessary, particularly in colonic intussusceptions and in older patients, because of the possibility of a malignant tumor. Some authors have recommended a selective approach to resection, depending on the site of intussusception, which influences the type of pathology [1,14]. Chang and colleagues [15] recommended operative reduction for small-bowel intussusceptions but not for colonic intussusceptions. Gupta and colleagues [16] reported resection in 70% of colonic intussusceptions. During last years a few selected cases of successful laparoscopicresection under colonoscopic guidance of symptomatic colonic lipomas have been reported [17,18]. The present case highlights the possibility of intussusception with an unusually benign cause, such as lipoma, when adult patients present with nonspecific abdominal symptoms and small bowel obstruction. Diagnosis can be determined radiologically or colonoscopically. CT imaging remain the methods of choice for studying abdominal lipomas, particularly those rising into the layers of the colonic wall. Surgical resection remains the treatment of choice and produces an excellent prognosis. Additional reports of intussusception in the adult population are needed to optimize the standard management for this uncommon disease.

## Consent

Written informed consent was obtained from the patient for publication of this case report and accompanying images.

## Abbreviations

CT: Computed tomography; MRI: Magnetic resonance imaging; CS: Colonoscopy; ECS: Enema contrast study; ND: Not described.

## Competing interests

The authors declare that they have no competing interests.

## Authors’ contributions

All of the authors were involved in the preparation of this manuscript. OM performed the operation and revised the manuscript. HH was an assistant surgeon and made substantial contributions to conception and design. LC described histological finding and was involved in drafting the manuscript. All authors read and approved the final manuscript.

## Pre-publication history

The pre-publication history for this paper can be accessed here:

http://www.biomedcentral.com/1471-2482/13/51/prepub
